# Shape and interaction decoupling for colloidal preassembly

**DOI:** 10.1126/sciadv.abm0548

**Published:** 2022-05-27

**Authors:** Lucia Baldauf, Erin G. Teich, Peter Schall, Greg van Anders, Laura Rossi

**Affiliations:** 1 Institute of Physics, University of Amsterdam, 1098XH Amsterdam, Netherlands.; 2 Applied Physics Program, University of Michigan, Ann Arbor, MI 48109,USA.; 3 Department of Physics, University of Michigan, Ann Arbor, MI 48109, USA.; 4 Department of Physics, Engineering Physics, and Astronomy, Queen’s University, Kingston, ON K7L3N6, Canada.; 5 Department of Chemical Engineering, Delft University of Technology, 2629 HZ Delft, the Netherlands.

## Abstract

Creating materials with structure that is independently controllable at a range of scales requires breaking naturally occurring hierarchies. Breaking these hierarchies can be achieved via the decoupling of building block attributes from structure during assembly. Here, we demonstrate, through computer simulations and experiments, that shape and interaction decoupling occur in colloidal cuboids suspended in evaporating emulsion droplets. The resulting colloidal clusters serve as “preassembled” mesoscale building blocks for larger-scale structures. We show that clusters of up to nine particles form mesoscale building blocks with geometries that are independent of the particles’ degree of faceting and dipolar magnetic interactions. To highlight the potential of these superball clusters for hierarchical assembly, we demonstrate, using computer simulations, that clusters of six to nine particles can assemble into high-order structures that differ from bulk self-assembly of individual particles. Our results suggest that preassembled building blocks present a viable route to hierarchical materials design.

## Teaser

Shape and interaction decoupling of cubic colloids allows the formation of ordered structures different from bulk assembly.

## INTRODUCTION

**Fig. 1.**
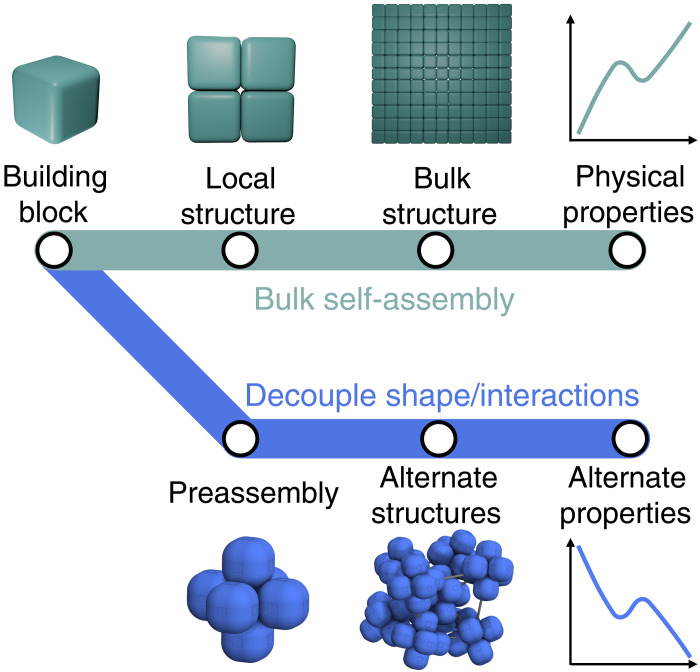
Schematic illustration of different pathways to hierarchical assembly. Without intervention, structure and material properties follow directly from building block attributes (green “subway line” above). Alternate material properties require an alternate route, such as preassembly (blue subway line) to create mesoscale structures that are not found in bulk.

A prevailing challenge in materials engineering is to produce materials with controlled, hierarchical structure at several length scales (*1*, *2*). Hierarchically structured materials can arise in one of two ways. The first is serendipitous and occurs if target structural motifs at multiple scales arise spontaneously from building blocks (*3*) with finely tuned attributes. Although there have been advances in inverse-design techniques for engineering building block attributes for target structures (*4*–*6*), these techniques have not yet been extended to hierarchical structures. The other way to achieve hierarchical structure is by intervening during the assembly process to decouple structural outcomes from building block attributes. Preassembling clusters of building blocks into nonbulk motifs provides an avenue to do this. For spherical building blocks, preassembly has been reported in (*7*, *8*) in which DNA-mediated and magnetic dipolar interactions were used to program hierarchical assembly. The hierarchical assembly observed in simulation (*9*, *10*) and experiment (*11*) in diverse system types suggests that hierarchical assembly can be achieved if appropriately preassembled building blocks can be made. However, for preassembly to serve as a generic route to hierarchical structure, preassembly protocols must exist for generic building blocks, and generic building blocks are shape-anisotropic. Preassembling shape-anisotropic building blocks into target motifs would appear to present a major challenge because shape is a strong, fundamental determinant (*12*, *13*) of structure in bulk assembly. Hence, for generic, shape-anisotropic building blocks, the key challenge for hierarchical assembly is to decouple elementary building block geometry from the structural motifs of intermediate-scale, preassembled building blocks. Achieving this decoupling is a prerequisite for preassembly as a viable route for hierarchical materials (see Fig. 1 for a schematic illustration).

Here, we demonstrate experimentally that we can induce colloidal shape-anisotropic building blocks to arrange in geometric structures that are not found in bulk self-assembly. The nonbulk geometric structures we create in experiment can be regarded as a form of “preassembly” that produces mesoscale building blocks with geometric arrangements that are decoupled from the attributes of the underlying microscopic building blocks. Using computer simulation, we show that these preassembled building blocks self-assemble to produce identical macroscopic structures that differ in their intermediate, mesoscale structure in a way that is dictated by the form of the preassembled building block.

We achieve shape decoupling by exploiting unique geometric effects that occur when particles are packed in confinement. Without confinement, the bulk self-assembly of shape-anisotropic colloids is driven by a subtle interplay of multiple forces (*13*, *14*), including steric effects, which are controlled by particle shape (*12*, *13*). In confinement, however, theoretical predictions (*15*) indicate that under idealized conditions, perfectly hard particles in perfectly hard confinement exhibit a range of structural organization that can be at odds with the particle symmetry and with bulk packing or assembly behavior. This confinement-induced deviation from bulk behavior occurs because the interaction of the building blocks with the container competes with the shape-driven steric repulsion among the building blocks, reducing the influence of shape anisotropy (*15*). This confinement-induced reduction in the influence of building block shape has the effect of decoupling the local order of the building blocks from their shape (*15*), which dominates their behavior in bulk (*12*, *13*). If this putative effect could be realized in experiment through some kind of confinement mechanism, then it would be possible to produce mesoscale building blocks within confinement whose structure breaks the emergent hierarchy that exists for free particles in bulk. Similar mechanisms are known to produce structural arrangement in biology; the packing and crowding of macromolecules in cells (*16*), the growth of cellular, bacterial, and viral aggregates (*17*–*19*), and blood clotting (*20*) are a few salient examples.

We demonstrate an analogous effect in synthetic colloids using a combination of shape-controlled synthesis and emulsification to confine small numbers of colloids into clusters that defy the arrangement tendencies of their shape and interactions in bulk (*21*–*24*). We show, using experiments and computer simulations, that compressing colloidal superballs with different degrees of sphericity in spherical confinement generates reproducible clusters whose structures are remarkably different from those found in the bulk. We confirm experimentally that these clusters are also formed by magnetic colloidal superballs with strong dipolar interparticle interactions, demonstrating that particle interaction and particle shape can be decoupled via spherical confinement. We also show via computer simulations that clusters of six to nine particles form higher-order assemblies whose structures differ from those observed in bulk assembly of similar particles. This work introduces a general principle that can be applied to other shape-anisotropic particles to further explore the development of novel materials via hierarchical assembly.

## RESULTS AND DISCUSSION

### Experimental system

**Fig. 2.**
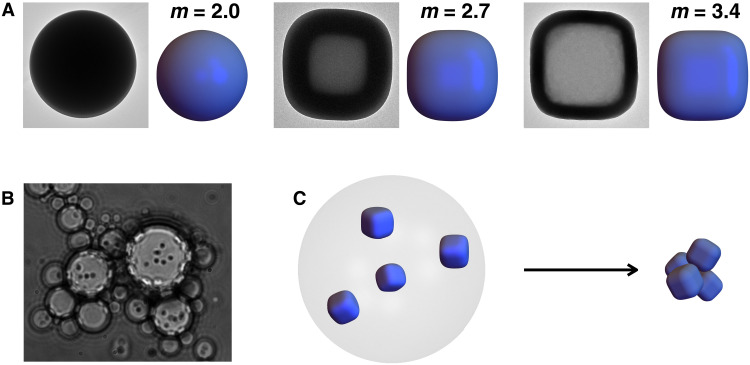
Preassembled building blocks are created from rounded silica cubes (superballs) compressed in emulsion droplets. (**A**) TEM images and models of superballs with shape parameter of 2.0 (left), 2.7 (middle), and 3.4 (right). (**B**) Light microscope image of the water-in-oil emulsion containing freely diffusing (spherical) particles. (**C**) Schematic illustration of the emulsion drying process that compresses the particles together to form a colloidal cluster.

Preassembly pathways were investigated using colloidal silica cubes with rounded edges. These particles can be modeled as “superballs,” described by ∣*x*/*a*∣*^m^* + ∣*y*/*a*∣*^m^* + ∣*z*/*a*∣*^m^* = 1, where *L* = 2*a* is the particle’s edge length and *m* is the shape parameter, a value that characterizes the roundness of the particle’s corners (see Materials and Methods for details on simulated particles). This shape is particularly interesting because it smoothly interpolates with *m* from a sphere at *m* = 2 to cubic particles with increasingly sharper corners as *m* → ∞. Here, we used silica particles with three distinct shape parameters: *m* = 2,2.7 and *m* = 3.4 (Fig. 2A). Shape parameters were determined using high-resolution transmission electron microscopy (TEM) images of single particles by fitting the particles edges with a superdisk equation [to account for the two-dimensional (2D) projections of the superballs in TEM images] (*14*). Polydispersity in *m* was calculated from the fits to be 4.4 and 4.9% for *m* = 2.7 and *m* = 3.4, respectively. Experimentally, silica superballs were prepared by growing layers of amorphous silica on the surface of cubic hematite particles, with *m* decreasing with the thickness of the silica layer, as reported in (*14*). Silica spheres, *m* = 2, with a size of 648 nm were prepared following the classical Stöber method (*25*), while silica spheres with a size of 1.2 μm were purchased from Bangs Laboratories. We preassembled these particles into small clusters using water-in-oil emulsions following the procedure reported by Cho *et al.* (*26*). This method allows for particles to freely diffuse inside the droplets (see Fig. 2B) during compression rather than adsorb at the interface affecting their orientations (*27*, *28*) and possibly influencing their final packing geometry. In a typical experiment, an aqueous silica dispersion was emulsified in hexadecane using Hypermer as a surfactant (see the Materials and Methods for details). The clusters were formed by compression of slowly evaporating water droplets as shown schematically in Fig. 2C. Once formed, particles within the clusters are held together by strong van der Waals forces, which are strong enough to allow drying and imaging of the clusters with a scanning electron microscope (SEM).

### Comparison to sphere clusters

To test the validity of the experimental clustering procedure, we first reproduced clusters of spherical particles using silica spheres. The results are shown in fig. S1. We find that the procedure is reproducible and that the clusters obtained are representative of the different geometries found by Cho *et al.* (*26*) for water-in-oil emulsions of silica spheres.

**Fig. 3.**
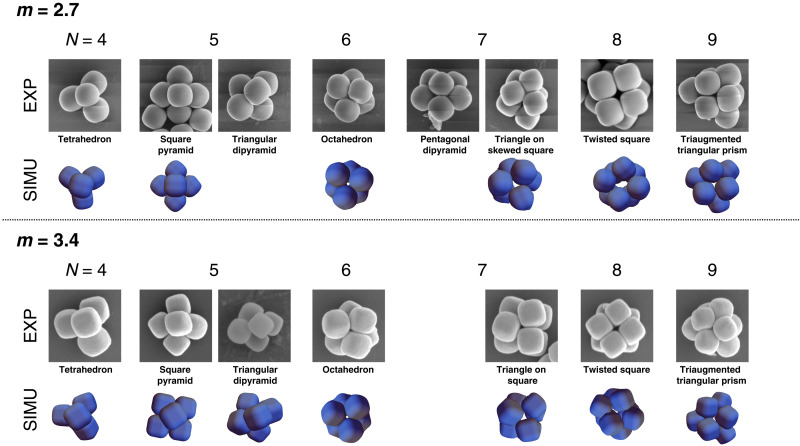
Experimental and simulated clusters of rounded cubes. SEM images of clusters generated from superballs with shape parameters *m* = 2.7 and *m* = 3.4, compared with simulated clusters of superballs with the same shape parameters. EXP, experiment; SIMU, simulation.

For shape parameters *m* = 2.7 and *m* = 3.4, the resulting packing configurations are shown in Fig. 3. Despite the superballs’ lower rotational symmetry, we find that, as with spheres, their packing is uniquely defined and differs from that of bulk assembly of particles with the same shape parameter. In bulk, the assembly of superballs can take the form of a *C*_0_ or *C*_1_ lattice depending on the *m* value of the particles (*21*–*23*). We find that we consistently obtain clusters whose geometries match those of the corresponding sphere clusters shown in fig. S1 and found by other experimental (*26*, *29*, *30*) and simulation works (*15*, *31*). This is unexpected because for *m* = 2.7 and *m* = 3.4, particles show clear facets (see Fig. 2A) and therefore might be expected to pack less similar to spheres and more similar to cubes. These expectations were also supported by a recent work by some of the authors showing differences in geometry for clusters formed by the platonic solids. These clusters were generated via computer simulations through compression of a spherical container (*15*), a method that can be directly related to the experimental evaporation of water-in-oil emulsion droplets that we are using. In their work, Teich *et al.* (*15*) showed that clusters of perfect cubes (*m* = ∞) present a common structure with clusters of spheres only for clusters of *N* = 4 and *N* = 5 particles. They found that clusters of higher numbers of perfect cubes are not similar to clusters of spheres according to a similarity metric comparing vectors of descriptive Steinhardt order parameters. Instead, these clusters tend to organize into layers with primarily orthorhombic, monoclinic, or cubic symmetries. Thus, these cluster structures are highly influenced by the cubic shape of their constituent particles. This coupling between shape and structure is in contrast to the decoupling found in this work for cubes with rounded edges (shape parameter *m* = 2.7 and *m* = 3.4).

For clusters of *N* = 5 particles (*m* = 2.7 and *m* = 3.4), we find degenerate structures: square pyramid and triangular dipyramid. Although experimental works on sphere clusters of both water-in-oil (*26*) and oil-in-water (*29*) emulsions have reported only triangular dipyramid clusters, we consistently find both geometries in our experiments with spherical particles (see fig. S1).

### Comparison to simulation

Experimental clusters were formed when evaporating water droplets slowly compressed the colloids, suggesting that the experimental clusters should be densely packed. To test this, we modeled the colloids as hard particles and simulated compression of small numbers of particles inside a spherical droplet. We computationally generated dense clusters of hard superballs with the experimental shape parameters *m* = 2.7 and *m* = 3.4 using isobaric Monte Carlo (MC) simulations identical in protocol to those described in *15*. Note that, in contrast to oil-in-water emulsions where particles would adhere to droplet surfaces, in water-in-oil emulsions, particles disperse within droplets. To capture this effect in our model, we encased our particles inside a spherical container and enforced confinement by rejecting trial particle moves if they resulted in any overlaps between particles and the surrounding spherical wall. We also rejected trial particle moves if they resulted in any overlaps between particles. We induced compression of the spherical container by exponentially increasing system pressure to a putatively high value during the simulation. We ran simulations of *N* = 4 to 9 particles for each shape parameter, and for every (*N*, *m*) state point, we ran 50 compression simulations. We chose the densest cluster achieved at each (*N*, *m*) state point for further analysis and comparison to experimental results (see the Materials and Methods).

A comparison between representative SEM images of clusters of superballs with *m* values of 2.7 and 3.4 and the corresponding clusters achieved via simulation is included in Fig. 3. (The robustness of simulation results for different values of *m* is summarized in fig. S3). We find good qualitative agreement between experiments and simulations. For each (*N*, *m*) state point, a majority (more than 80%) of our 50 replicate simulations resulted in approximately the same final cluster structure, and this structure (the densest of which is shown in Fig. 3) approximately matches experimental results. A notable exception is the case of *N* = 5. For (*N*, *m*) = (5, 2.7), we find only the square pyramid in our simulations (with all four base particles aligned face to face in 44 replicates or with one base particle misaligned in the remaining replicates). For (*N*, *m*) = (5, 3.4), however, we find a skewed square pyramid in only 24 of 50 replicate simulations and an approximate triangular bipyramid in the remaining 26 of 50 simulations. The triangular bipyramid is the denser final structure, since the density of the densest triangular bipyramid is larger than the density of the densest square pyramid by ≈3.7 × 10^−4^.

**Fig. 4.**
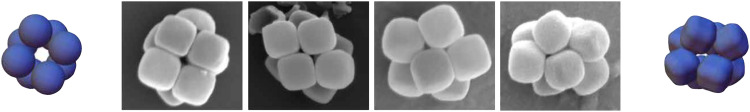
Clusters of eight superballs show diverse arrangements. SEM images of isomeric clusters of eight particles with shape parameter *m* = 3.4. The model on the left shows a twisted-square structure as obtained with spheres, and the model on the right shows a compressed structure similar to a snub disphenoid as obtained with superballs with *m* = 3.4.

For *N* = 8, the prevalent structure found experimentally for superballs with shape parameters *m* = 2.7 and *m* = 3.4 is the twisted square (Fig. 3) that was also found in spheres in the experiments by Cho *et al.* (*26*) and simulations by Teich *et al.* (*15*). In the computer simulations of particles with the same shape parameters (also in Fig. 3), we obtain clusters with a compressed twisted-square structure. The same compressed structures were also present in experiments, and although less abundant than the twisted-square clusters, they were found in all samples. Figure 4 shows the compressed structures as a transition between the twisted-square structure and a snub disphenoid, a structure that minimizes the second moment of the mass distribution. This structure is largely found in clusters of spheres obtained from oil-in-water emulsion droplets of uncharged particles (*29*). It seems that although less favorable than the twisted square, the intermediate structures that were also predicted by our computer simulations are accessible by our systems. Computer simulations of particles with higher shape parameters (detailed in the next section and fig. S3) show that for clusters with *N* = 8 particles, there is a transition from twisted square for *m* = 2 to snub disphenoid for *m* = 2.7 and *m* = 3.4 and back to twisted square for *m* = 6 and *m* = 8. This transition is solely dependent on the shape parameter. Since the transition is shape dependent, we can hypothesize that the presence of isomers in the experiments is attributable to particle polydispersity in shape.

### Simulation of higher *m* values

To further explore the importance of particle shape in determining cluster geometry, we performed computer simulations of particles with higher *m* values, specifically *m* = 6 and *m* = 8. Particles with such high shape parameters are not accessible using hematite particles and are in general more difficult to obtain experimentally in the micrometer size range. The results are reported in fig. S3. We find that, despite sharper particle edges, clusters of superballs with *m* = 6 and *m* = 8 show densest structures that are equivalent to those already found for lower *m* values. As mentioned above, of both *m* = 6 and *m* = 8, clusters with *N* = 8 particles show the twisted-square geometry most similar to that of clusters of spheres, although for *m* = 6 and *m* = 8, the two four-particle layers forming the clusters are off-centered by angles less than 45°.

### Clusters of magnetic superballs

**Fig. 5.**
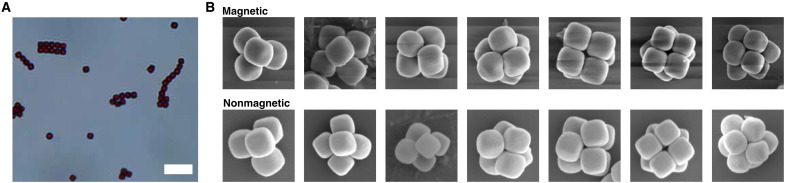
Geometric arrangement of superball clusters is driven by number of particles, not particle interactions. (**A**) Optical microscope image of the magnetic cubes forming 2D dipolar structures in a zero-field environment. Scale bar, 8 μm. (**B**) Representative SEM images of clusters prepared using magnetic (top row) and nonmagnetic (bottom row) superball particles. For all clusters, we see consistent geometries, which depend only on the number of constituent particles.

While packing considerations are clearly important to determine the final resulting cluster geometry, the question remains whether other factors, such as interparticle interactions, play a role in influencing the final structure. Recent computer simulations suggest that for magnetic particles with *m* = 4, the packing is still dominated by geometry rather than dipolar interaction (*8*). To test this result experimentally, we prepared clusters of magnetic particles with strong dipolar interparticle interactions. We used core-shell hematite-silica particles with comparable shape parameters prepared as described in (*32*). The procedure to prepare the magnetic core-shell particles is identical to that used for the preparation of the nonmagnetic particles, with only one difference: The internal magnetic hematite core is not dissolved once the silica shell has been deposited on its surface. The resulting particles have a superball shape, an external silica surface identical to that of the silica superballs, and a permanent magnetic dipole moment strong enough (μ_p_ ≈ 3 × 10^−15^ Am^2^) to induce dipolar structure formation even in the absence of an external magnetic field (*32*). An example of these dipolar structures can be seen in the optical microscope image in Fig. 5A.

We find that clusters made using magnetic superballs again have the same geometry as those formed by (nonmagnetic) silica superballs, confirming that shape is the crucial parameter in determining the final cluster geometry and that the experimental spherical confinement procedure is capable of decoupling interparticle interaction from resultant cluster structure.

### Hierarchical self-assembly

Our experiments produced rigidly structured clusters of colloids held together by Van der Waals forces. To test whether the clusters of nonmagnetic colloids produced in experiment provide viable candidates for hierarchical self-assembly, we modeled the experimentally produced clusters as rigid collections of colloids in MC simulations. We performed simulations of *N*_c_ = 343 clusters and observed the self-assembly of ordered structures over a range of fixed densities, using the hard-particle MC (HPMC) plugin (*33*) for HOOMD-blue (*34*). We used a standard NVT MC simulation protocol that has been widely adopted in other investigations [e.g., (*6*, *13*, *35*, *36*)].

**Fig. 6.**
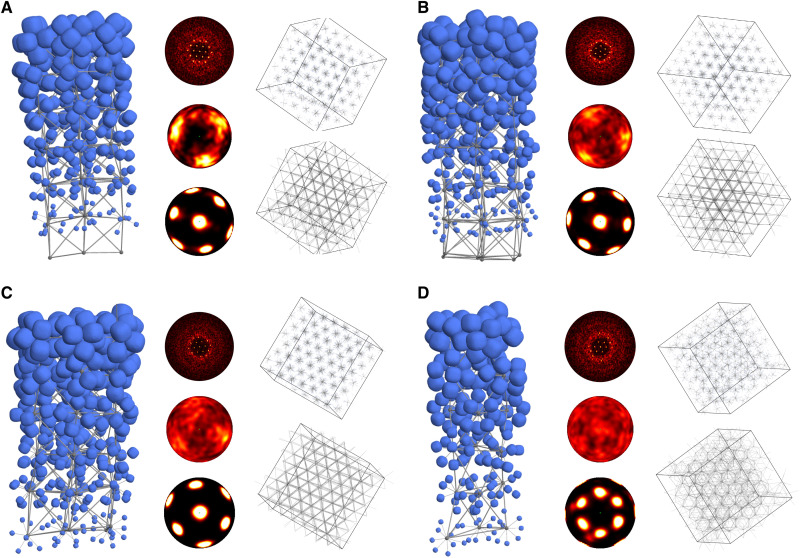
Preassembled superball clusters form hierarchical assemblies. The self-assembled systems shown here consist of *m* = 2.7 superball clusters with centers arranged in a BCC structure for (**A**) *N* = 6, ϕ = 0.4; (**B**) *N* = 7, ϕ = 0.4; and (**C**) *N* = 8, ϕ = 0.42; and an HCP structure for (**D**) *N* = 9, ϕ = 0.4. Each panel displays a section of the self-assembled structure on the left, with a gradient of superball sizes shown for ease of visualization of the underlying structure. Diffraction patterns calculated for all superballs are displayed in the top center of each panel, showing ordering at long range (small *k*) and weaker ordering at short range (large *k*). Diffraction patterns are oriented to show the threefold rotational symmetry of each structure. Two types of bond-orientational order diagrams are shown beneath each diffraction pattern. The first, located in the middle center of each panel, shows the orientational distribution of all bonds between cluster centers and their respective superballs. These bonds are also shown in the simulation image in the top right of each panel. The second, located in the bottom center of each panel, shows the orientational distribution of all bonds between neighboring cluster centers. These bonds are shown in the simulation image in the bottom right of each panel and indicate the crystal lattice vectors of the self-assembled clusters. Note that in all cases, comparison of the two bond-orientational order diagrams indicates that the cluster orientations are not randomly distributed throughout the self-assembled structure. Rather, the cluster orientations (and thus bonds between cluster centers and their superballs) appear to lie roughly either along a subset of the crystal lattice vectors (A to C) or in a complementary (yet noisy) fashion to the crystal lattice vectors (D). In all assembly snapshots, the cluster centers are also shown as gray spheres, for clarity.

In contrast to the structures typically observed in the self-assembly of cubic monomers (*14*, *21*–*24*), we found that superball clusters self-assembled into bulk BCC or HCP structures, structures more typical of the self-assembly of monomers of other shapes. We also observed that preassembled clusters self-assembled at lower densities than typically observed for monomer self-assembly. For example, we observed *N* = 6,8,9 clusters assembled hierarchically at packing densities as low as 38% and *N* = 7 at 40%, whereas monomer self-assembly is more typically observed at densities of 50% or more (*23*). Figure 6 shows examples of these self-assembled structures for clusters consisting of *N* = 6,7,8,9 superballs with *m* = 2.7.

We analyze the structures resulting from self-assembly simulations using simulated diffraction images and bond order diagrams following established protocols [e.g., (*6*, *12*, *36*)]. The hierarchical nature of the self-assembly is most clearly evident in the contrast between the low– and high–wave number behavior in the simulated diffraction images (top middle inset in each panel in Fig. 6). As in other studies of hard-particle crystallization [e.g., (*6*, *12*, *36*)], diffraction peaks at low wave number are clear indicators of well-defined, long-range order. Our results here show the BCC (Fig. 6, A to C) or HCP (Fig. 6D) arrangement of cluster centers. The ordered arrangement of cluster centers is also apparent in the bond order of cluster centers (bottom middle inset in each panel in Fig. 6). In contrast, the large wave number ring reflects a lack of sharp short-range order of the cluster orientations. Bond order diagrams for within cluster particles (center middle inset in each panel in Fig. 6) show that cluster orientations exhibit varying degrees of weak alignment with lattice directions. Crucially, we consistently observed this form of bulk self-assembly for clusters containing different numbers of particles, hence different local symmetries. Because of the geometric arrangement of the particles within clusters, clusters of different sizes have distinct organization. This means that although we observed consistent long-range order in our cluster assemblies, the short-range order is considerably different across different clusters. We also note that, in addition to the change in structural order we observe for the hierarchical assembly of clusters, the >10% reduction in required density for the self-assembly of preassembled clusters versus monomers suggests that preassembly could be a route to lower density structures.

We demonstrated that the emulsification of shape-anisotropic colloids produces clusters with geometric arrangements not observed in the bulk self-assembly or packing of similarly shaped particles (*14*, *21*–*24*), that are at odds with the underlying particle symmetry. We found that treating these clusters as preassembled building blocks yields hierarchically structured self-assembly in computer simulations. Our experimental findings that similar clustering effects persist for particles, which are shape anisotropic and have magnetic interactions, demonstrate that our approach can be used to suppress the effects of both shape and interaction. Collectively, our results demonstrate that our emulsification method produces a form of shape and interaction decoupling that can be exploited as a route to the general preassembly of building blocks for the design of hierarchically structured materials.

The experiments we performed here involved clusters of up to nine building blocks. Simulations predict that similar effects can be achieved in clusters with dozens of building blocks (*15*), and experiments suggest that nonbulk behavior can be produced in clusters containing hundreds of thousands of building blocks (*37*). These results suggest that the preassembly approach we demonstrate here can extend to building block geometries, forms of interaction, and cluster sizes well beyond those we studied. Note that if clusters have complete freedom to rearrange, in bulk assembly, then they will rearrange to give nonhierarchical bulk structure. To form hierarchical structure at large scales like we observed here, effective preassembly must produce building blocks where the forces that maintain the preassembled building blocks are stronger than those that drive the subsequent bulk assembly.

Moving beyond the present proof of principle requires achieving scale and generalization. For scale, we note that prior work shows that emulsification can be followed by the successful separation of clusters, usually via density gradient centrifugation (*29*). In terms of generalization, on the theory side, building blocks with geometries of the sort presented here can be incorporated into inverse design approaches. Preassembled building blocks that are sufficiently small to maintain Brownian motion can be included in an inverse design approach like digital alchemy (*4*, *6*). For larger building blocks that become granular, preassembled building blocks formed from densely packed clusters could be used to generate “mutations” in evolutionary design approaches (*5*). On the experimental side, our results showing that similar effects can be produced in particles with shape anisotropy and a magnetic core suggest that further application of this method could be immediately refined to produce preassembled building blocks with magnetic patches (*8*). Additional experiments with differently shaped particles will yield colloidal clusters with unconvenntional geometric complexity and specific surface functionalization, forming the basis for further development of hierarchically structured materials.

## MATERIALS AND METHODS

### Preparation of silica superballs

Silica superballs with different shape parameters were prepared using the procedure described in a previous work by some of the authors (*14*). Silica spheres (superballs with *m* = 2) were prepared following the well known Stöber method (*25*).

### Preparation of superball clusters

Colloidal clusters were prepared from water-in-oil emulsions following a modified version of Cho *et al.*’s procedure (*26*). The oil phase was prepared by dissolving 0.0144 g of Hypermer 2296 [2266-LQ-(MV), donated by Croda] in 4.808 g of hexadecane (ReagentPlus 99%, Sigma-Aldrich). The water phase consisted of 780 μl of particle dispersion (silica or hematite-silica particles) at an approximate concentration of 7.5 × 10^−9^ colloids/ml. The two phases were emulsified using a Silverson L5M-A emulsifier rotating first at 8000 rpm for 40 s and then at 9500 rpm for an additional 40 s. After emulsification, the vial containing the emulsion was placed in a heated glycerol bath at a constant temperature of 100°C. The emulsion was magnetically or mechanically stirred throughout the whole drying process, which typically lasted 1 hour. The evaporation process was monitored in time by optical microscopy observations. The newly formed clusters were then washed by sedimenting and redispersing in hexane containing a low concentration [≤0.1 weight % (wt %)] of Hypermer. Clusters can then be stored in hexane or dried and redispersed in a 0.1 wt % SDS aqueous solution.

### Electron microscopy

Particle size and shape were determined by TEM (Philips TECNAI12). Samples were prepared by drying drops of diluted dispersions on carbon and polymer-coated copper grids. Cluster geometries were studied by SEM (FEI Helios NanoLab 600). Samples were prepared by drying drops of dispersions on carbon and polymer-coated copper grids or small pieces of silicon wafer, which were then mounted on 15-mm SEM stubs using conductive tape. All SEM samples were sputter-coated (Leica EM ACE600) with a layer of 5-nm Cr and 5-nm Au.

### Optical microscopy

Emulsions and clusters were imaged using a Zeiss Axio Imager A1 upright microscope with a Plan Neofluar 100× oil immersion objective. Dispersions were placed in flat rectangular borosilicate glass capillaries (CM Scientific 0.2 mm by 2 mm by 4 cm), sealed to a microscope slide with wax.

### Simulations of colloidal clusters

We computationally generated dense clusters of superballs via isobaric MC simulations using spherical confinement, identically to the protocol described in *15*. We used the HPMC (*33*) method in the open-source simulation toolkit HOOMD-blue (*34*, *38*). The computational workflow and data management were facilitated by the signac data management framework (*39*, *40*). We visualized our results using the software package Injavis (*41*). For each (*N*, *m*) state point, we placed *N* superballs with shape parameter *m* inside a sphere. We rejected trial particle translations or rotations if they resulted in any particle overlaps or overlaps between particles and the encasing sphere. We enforced increasing spherical confinement by raising dimensionless pressure exponentially from a minimum value of 0.1 to a maximum value of 500 in 10^4^ steps. Dimensionless pressure is defined as p^*^ = β*pl*^3^, where *p* is pressure and *l* = *L* is the superball edge length as defined in Results and Discussion. At each pressure value, we allowed the system to equilibrate for 10^3^ HPMC time steps. Throughout each simulation, we tuned particle translation and rotation move sizes, as well as system volume move sizes, to maintain move acceptance ratios of approximately 0.2.

We ran simulations of *N* = 4 to 9 particles for each shape parameter, and for every (*N*, *m*) state point, we ran 50 independent compression simulations. We calculated the densities of each resultant cluster according to ϕ = *Nv*_p_/*V*, where *v*_p_ is the volume of a single particle and *V* is the volume of the spherical container, and chose the densest cluster (with highest ϕ) for further analysis and comparison to experimental results.

We characterized each cluster structure according to the geometry and topology of its network of virtual “bonds” between particle centers, built by calculating radial distribution functions (RDFs) for each cluster and identifying nearby particles according to RDF peaks. Clusters were primarily identified by qualitative comparison to a library of cluster structures built in previous work (*15*) via numerical comparison to spherical code solutions and visual examination of bond order. We also visually examined the orientations of the particles in each superball cluster in this work and incorporated that information into cluster descriptions as appropriate throughout Results and Discussion.

We approximated each superball, parameterized by (*L*, *m*), as a spherocube or a cube with a sphere swept around its edges, parameterized instead by (σ, *d*). σ is the side length of the cube, and *d* is the diameter of the sweeping sphere. We used the approximating spherocubes as the particle shapes in our simulations because the spheropolyhedron overlap check was already implemented in HPMC and because spherocubes are very good approximations of superballs. To generate each approximation, we followed the prescription laid out in appendix C of. (*42*); we refer the interested reader to that paper for more details. Briefly, we found (σ, *d*) such that the corresponding spherocube had the minimal Hausdorff distance from the superball defined by each (*L*, *m*) value of interest. The Hausdorff distance *d*(*A*, *B*) between bodies *A* and *B* is defined as

d(A,B)=max{d′(A,B),d′(B,A)}

$$d\prime (A,B)\equiv \text{ma}x_{x\in A}\text{mi}n_{y\in B}|x-y|$$

Calculation of the Hausdorff distance between a superball and spherocube, *d*(*L*, *m*, σ, *d*), is straightforward, as it is only necessary that one finds the maximal distance over three special pairs of {*x*, *y*} points. (The three pairs lie along the two-, three-, and fourfold symmetry axes of the aligned spherocube/superball pair, respectively.) We set *L* = 1 and found the following minimal Hausdorff distances between each (*L*, *m*) superball and its optimally matching (σ, *d*) spherocube

d(1,2.7,0.209,0.795)≈0.0019

d(1,3.4,0.347,0.658)≈0.0024

d(1,6.0,0.604,0.401)≈0.0024

d(1,8.0,0.696,0.308)≈0.0021

These were the spherocube parameters we used in our simulations.

### Hierarchical assembly

We tested for the possibility of hierarchical self-assembly by treating simulated colloidal clusters as hard, preassembled building blocks that we simulated using the HPMC (*33*) method in the open-source simulation toolkit HOOMD-blue (*34*,*38*). We initialized systems of *N* = 343 clusters at low density, which we thermalized for 10^5^ MC sweeps, and compressed systems to target packing fractions of 38% and above more than 5 × 10^6^ MC sweeps. We examined final simulation snapshots for structural order by examining simulated diffraction patterns and bond order diagrams via the software package Injavis (*41*). We did not observe conclusive evidence of self-assembly on the time scale of our simulations for clusters of particles with *m* = 3.4. For *m* = 2.7, we observed hierarchical self-assembly of clusters of *N* = 6,7,8,9 particles.

## Supplementary Materials

### This PDF file includes:

Supporting Data Figs. S1 to S3 References
