# Supplementary material for: Shape and interaction decoupling for colloidal preassembly
Source: Sci Adv. 2022 May 27;8(21):eabm0548. doi: 10.1126/sciadv.abm0548 (PMC9140958; doi:10.1126/sciadv.abm0548)
Supplement: Supplementary file 1 — Supporting Data Figs. S1 to S3 References [file sciadv.abm0548_sm.pdf]

Science Advances
AAAS

Supplementary Materials for

Shape and interaction decoupling for colloidal preassembly

Lucia Baldauf *et al*.

Corresponding author: Greg van Anders, gva@queensu.ca; Laura Rossi, l.rossi@tudelft.nl

Sci. Adv. **8**, eabm0548 (2022)
DOI: 10.1126/sciadv.abm0548

This PDF file includes:

Supporting Data
Figs. S1 to S3
References

Supporting data

Sphere Clusters

To test the validity of the experimental procedure we reproduced clusters of spherical particles using two different samples of silica spheres: one with diameter $d = 466$ nm synthesized by us and one with diameter $d = 1.2$ µm purchased from Bangs Laboratories Inc. The collective results are reported in Figure S1. The results obtained show that we have successfully reproduced the procedure reported by Cho *et al.* [26]. In our experiments for clusters with $N = 5$ components we find, however, both a triangular dypiramid, which structure minimizes the second-moment of the mass distribution, and a square pyramid which has not been reported so far in emulsion experiments.

The experimental observations match very well with the clusters obtained by computer simulations, with the exception of $N = 8$, where simulations show a twisted square configuration, but experiments find a snub disphenoid, and $N = 7$, where no match was found between our experiments and simulations. In our experiments we observe only one of the isomers (pentagonal dipyramid) reported for clusters generated from w-i-o emulsions [26], whereas the other isomer (tetramer-on-trimer) is predicted by our model.

In the case of $N = 5$, 30 of our 50 replica simulations resulted in the square pyramid, while the remaining 20 resulted in configurations resembling triangular bipyramids. We found the densest cluster to be a square pyramid, but density distributions for the square pyramid and triangular bipyramid structures were approximately equal, and the density of the densest square pyramid structure was only larger than the density of the densest triangular bipyramid structure by $\approx 1.8 \times 10^{-6}$. It is known that the $N = 5$ spherical code, the densest configuration of equal radius circles on the surface of a sphere, has a continuum of solutions between the square pyramid and the triangular bipyramid [43]. Authors of Ref. [43] found that, for 5 spheres packing around a central smaller sphere, the square pyramid is entropically favorable over the triangular bipyramid, as it results in the highest vibrational freedom for the particles. Thus, the degeneracy that we see in these structures, both in experiments

and [illegible]

simu

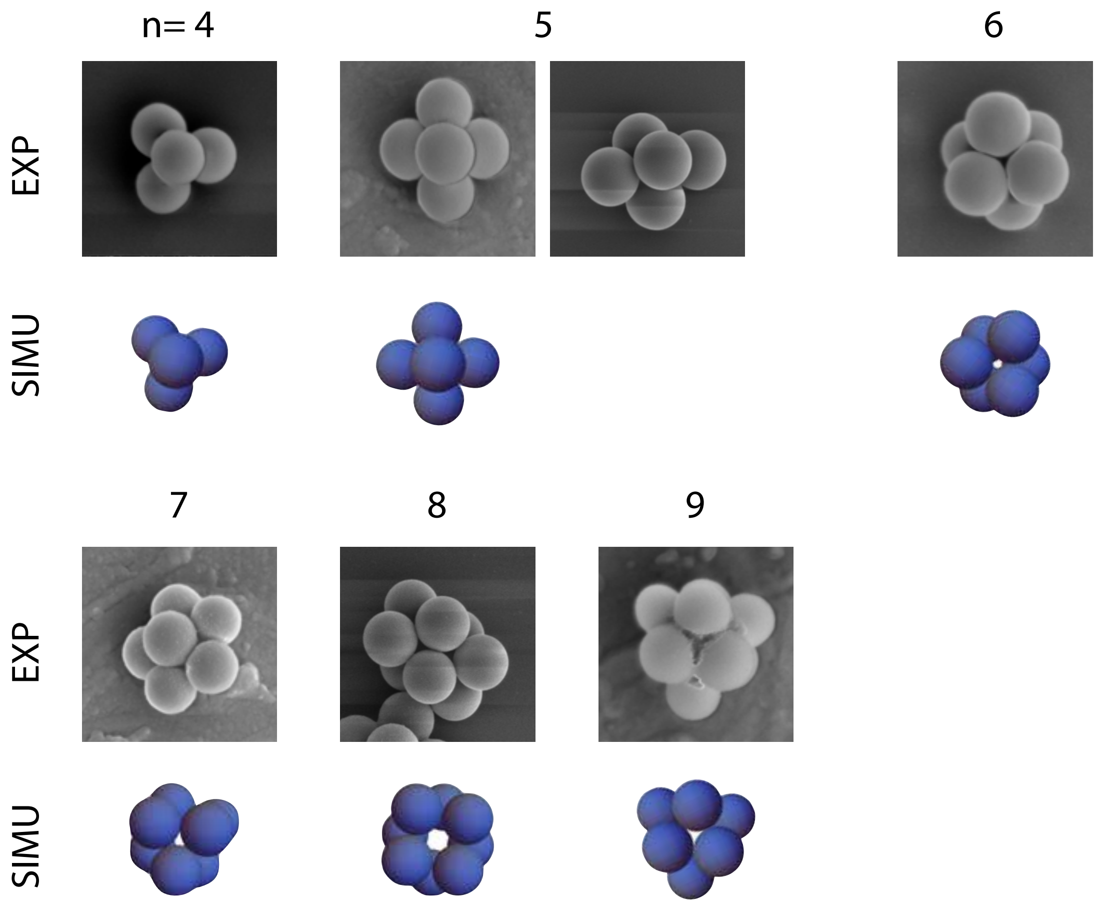

Figure S1: **Evaporating emulsion droplets of silica spheres generate densely packed clusters.** SEM images of clusters from water-in-oil emulsions obtained using spherical particles ($d = 648$ nm and 1.2 µm) and computer simulations of clusters with the same number of constituent spherical particles.

Su

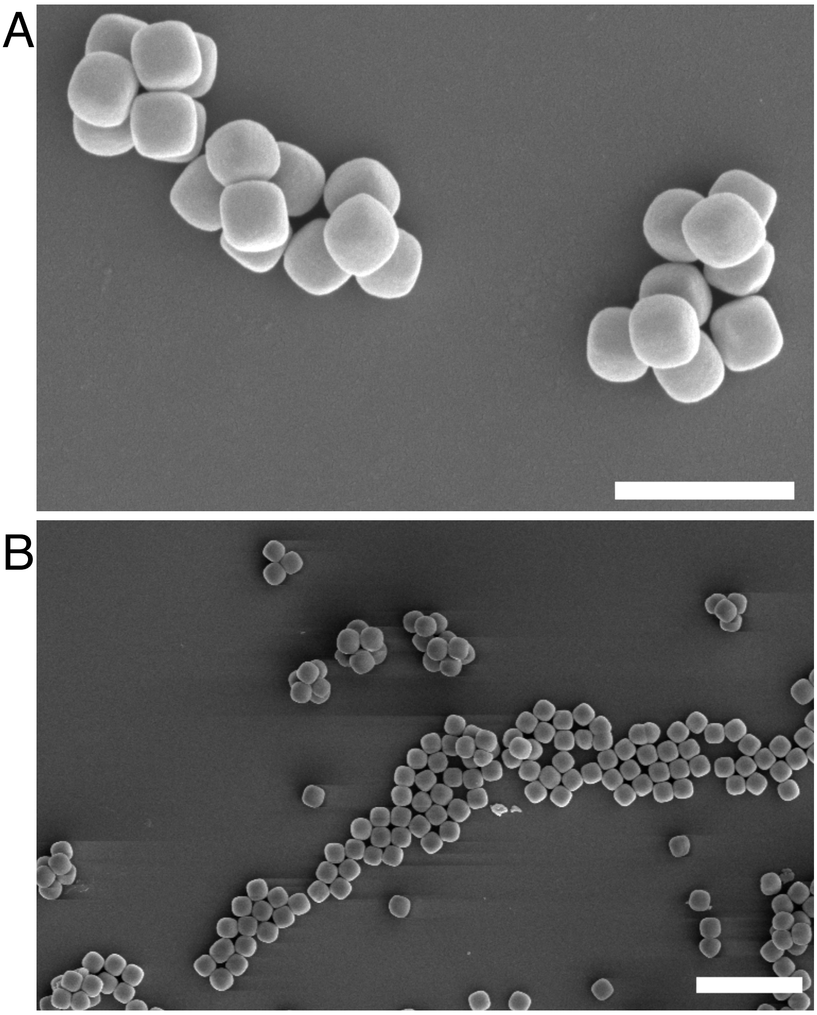

Figure S2: **Evaporating emulsion droplets of silica superballs produce diverse cluster sizes.** Low resolution SEM images of clusters. Scale bars 2μm (A) and 5μm (B)

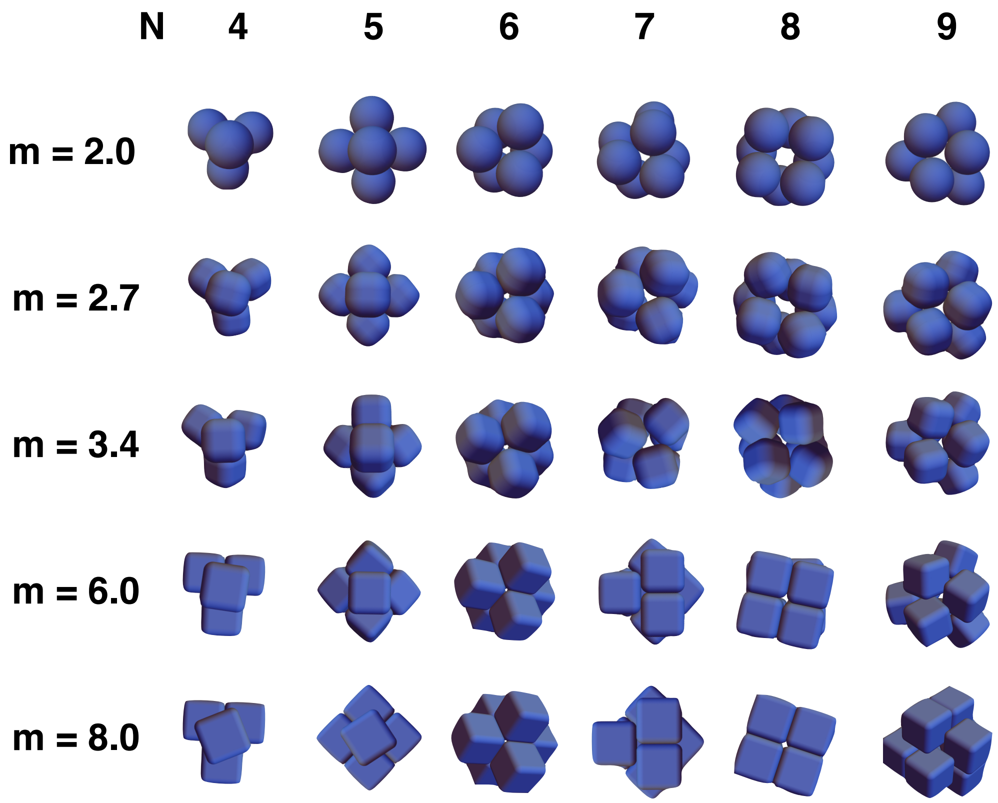

Figure S3: **Dense packing of spheres, cuboids, and shapes in between, produces consistent cluster arrangements across particle shapes.** Clusters generated by computer simulation for superballs with various m values.

REFERENCES AND NOTES

1. R. Lakes, Materials with structural hierarchy. *Nature* **361**, 511–515 (1993).

2. P. Fratzl, R. Weinkamer, Nature's hierarchical materials. *Prog. Mater. Sci.* **52**, 1263–1334 (2007).

3. S. C. Glotzer, M. J. Solomon, Anisotropy of building blocks and their assembly into complex structures. *Nat. Mater.* **6**, 557–562 (2007).

4. G. van Anders, D. Klotsa, A. S. Karas, P. M. Dodd, S. C. Glotzer, Digital alchemy for materials design: Colloids and beyond. *ACS Nano* **9**, 9542–9553 (2015).

5. M. Z. Miskin, H. M. Jaeger, Adapting granular materials through artificial evolution. *Nat. Mater.* **12**, 326–331 (2013).

6. Y. Geng, G. van Anders, P. M. Dodd, J. Dshemuchadse, S. C. Glotzer, Engineering entropy for the inverse design of colloidal crystals from hard shapes. *Sci. Adv.* **5**, eaaw0514 (2019).

7. É. Ducrot, M. He, G.-R. Yi, D. J. Pine, Colloidal alloys with preassembled clusters and spheres. *Nat. Mater.* **16**, 652–657 (2017).

8. J. G. Donaldson, P. Schall, L. Rossi, Magnetic coupling in colloidal clusters for hierarchical self-assembly. *ACS Nano* **15**, 4989–4999 (2021).

9. M. B. Zanjani, I. C. Jenkins, J. C. Crocker, T. Sinno, Colloidal cluster assembly into ordered superstructures via engineered directional binding. *ACS Nano* **10**, 11280–11289 (2016).

10. M. B. Zanjani, J. C. Crocker, T. Sinno, Self-assembly with colloidal clusters: Facile crystal design using connectivity landscape analysis. *Soft Matter* **13**, 7098–7105 (2017).

11. M. He, J. P. Gales, É. Ducrot, Z. Gong, G.-R. Yi, S. Sacanna, D. J. Pine, Colloidal diamond. *Nature* **585**, 524–529 (2020).

12. G. van Anders, N. K. Ahmed, R. Smith, M. Engel, S. C. Glotzer, Entropically patchy particles: Engineering valence through shape entropy. *ACS Nano* **8**, 931–940 (2014).

13. G. van Anders, D. Klotsa, N. K. Ahmed, M. Engel, S. C. Glotzer, Understanding shape entropy through local dense packing. *Proc. Natl. Acad. Sci. U.S.A.* **111**, E4812–E4821 (2014).

14. L. Rossi, V. Soni, D. J. Ashton, D. J. Pine, A. P. Philipse, P. M. Chaikin, M. Dijkstra, S. Sacanna, W. T. M. Irvine, Shape-sensitive crystallization in colloidal superball fluids. *Proc. Natl. Acad. Sci. U.S.A.* **112**, 5286–5290 (2015).

15. E. G. Teich, G. van Anders, D. Klotsa, J. Dshemuchadse, S. C. Glotzer, Clusters of polyhedra in spherical confinement. *Proc. Natl. Acad. Sci. U.S.A.* **113**, E669–E678 (2016).

16. R. J. Ellis, Macromolecular crowding: Obvious but underappreciated. *Trends Biochem. Sci.* **26**, 597–604 (2001).

17. T. Hayashi, R. W. Carthew, Surface mechanics mediate pattern formation in the developing retina. *Nature* **431**, 647–652 (2004).

18. J. A. Aström, M. Karttunen, Cell aggregation: Packing soft grains. *Phys. Rev. E* **73**, 062301 (2006).

19. C. P. Gerba, W. Q. Betancourt, Viral aggregation: Impact on virus behavior in the environment. *Environ. Sci. Technol.* **51**, 7318–7325 (2017).

20. D. B. Cines, T. Lebedeva, C. Nagaswami, V. Hayes, W. Massefski, R. I. Litvinov, L. Rauova, T. J. Lowery, J. W. Weisel, Clot contraction: Compression of erythrocytes into tightly packed polyhedra and redistribution of platelets and fibrin. *Blood* **123**, 1596–1603 (2014).

21. Y. Jiao, F. H. Stillinger, S. Torquato, Optimal packings of superballs. *Phys. Rev. E* **79**, 041309 (2009).

22. Y. Jiao, F. H. Stillinger, S. Torquato, Erratum: Optimal packings of superballs [Phys. Rev. E **79**, 041309 (2009)]. *Phys. Rev. E* **84**, 069902 (2011).

23. R. Ni, A. P. Gantapara, J. de Graaf, R. van Roij, M. Dijkstra, Phase diagram of colloidal hard superballs: From cubes via spheres to octahedra. *Soft Matter* **8**, 8826 (2012).

24. J.-M. Meijer, A. Pal, S. Ouhajji, H. N. W. Lekkerkerker, A. P. Philipse, A. V. Petukhov, Observation of solid-solid transitions in 3D crystals of colloidal superballs. *Nat. Commun.* **8**, 14352 (2017).

25. W. Stober, A. Fink, E. Bohn, Controlled growth of monodisperse silica spheres in the micron size range. *J. Colloid Interface Sci.* **26**, 62–69 (1968).

26. Y.-S. Cho, G.-R. Yi, S.-H. Kim, D. J. Pine, S.-M. Yang, Colloidal clusters of microspheres from water-in-oil emulsions. *Chem. Mater.* **17**, 5006–5013 (2005).

27. G. Soligno, M. Dijkstra, R. van Roij, Self-assembly of cubic colloidal particles at fluid–fluid interfaces by hexapolar capillary interactions. *Soft Matter* **14**, 42–60 (2018).

28. G. Soligno, M. Dijkstra, R. van Roij, Self-assembly of cubes into 2D hexagonal and honeycomb lattices by hexapolar capillary interactions. *Phys. Rev. Lett.* **116**, 258001 (2016).

29. V. N. Manoharan, M. T. Elsesser, D. J. Pine, Dense packing and symmetry in small clusters of microspheres. *Science* **301**, 483–487 (2003).

30. V. Manoharan, D. Pine, Building materials by packing spheres. *MRS Bull.* **29**, 91–95 (2004).

31. E. Lauga, M. P. Brenner, Evaporation-driven assembly of colloidal particles. *Phys. Rev. Lett.* **93**, 238301 (2004).

32. L. Rossi, J. G. Donaldson, J.-M. Meijer, A. V. Petukhov, D. Kleckner, S. S. Kantorovich, W. T. M. Irvine, A. P. Philipse, S. Sacanna, Self-organization in dipolar cube fluids constrained by competing anisotropies. *Soft Matter* **14**, 1080–1087 (2018).

33. J. A. Anderson, M. Eric Irrgang, S. C. Glotzer, Scalable Metropolis Monte Carlo for simulation of hard shapes. *Comput. Phys. Commun.* **204**, 21–30 (2016).

34. J. A. Anderson, C. D. Lorenz, A. Travesset, General purpose molecular dynamics simulations fully implemented on graphics processing units. *J. Comput. Phys.* **227**, 5342–5359 (2008).

35. P. F. Damasceno, M. Engel, S. C. Glotzer, Crystalline assemblies and densest packings of a family of truncated tetrahedra and the role of directional entropic forces. *ACS Nano* **6**, 609–614 (2012).

36. P. F. Damasceno, M. Engel, S. C. Glotzer, Predictive self-assembly of polyhedra into complex structures. *Science* **337**, 453–457 (2012).

37. B. de Nijs, S. Dussi, F. Smallenburg, J. D. Meeldijk, D. J. Groenendijk, L. Filion, A. Imhof, A. van Blaaderen, M. Dijkstra, Entropy-driven formation of large icosahedral colloidal clusters by spherical confinement. *Nat. Mater.* **14**, 56–60 (2015).

38. J. Glaser, T. D. Nguyen, J. A. Anderson, P. Lui, F. Spiga, J. A. Millan, D. C. Morse, S. C. Glotzer, Strong scaling of general-purpose molecular dynamics simulations on GPUs. *Comput. Phys. Commun.* **192**, 97–107 (2015).

39. C. S. Adorf, P. M. Dodd, V. Ramasubramani, S. C. Glotzer, Simple data and workflow management with the signac framework. *Comput. Mater. Sci.* **146**, 220–229 (2018).

40. C. S. Adorf, P. M. Dodd, V. Ramasubramani, B. Swerdlow, J. Glaser, B. Dice, csadorf/signac v0.9.2 (2017).

41. M. Engel (2021). INJAVIS – INteractive JAva VISualization (0.82). Zenodo. https://doi.org/10.5281/zenodo.4639570

42. M. Marechal, U. Zimmermann, H. Löwen, Freezing of parallel hard cubes with rounded edges. *J. Chem. Phys.* **136**, 144506 (2012).

43. C. L. Phillips, E. Jankowski, M. Marval, S. C. Glotzer, Self-assembled clusters of spheres related to spherical codes. *Phys. Rev. E* **86**, 041124 (2012).
